# Laparoscopic Management of Acute Pancreatitis Secondary to Rapunzel Syndrome

**DOI:** 10.1155/2016/7638504

**Published:** 2016-04-10

**Authors:** Bijan Koushk Jalali, Alperen Bingöl, Ashraf Reyad

**Affiliations:** Department of Surgery, The Johns Hopkins Hospital, 1800 Orleans Street, Baltimore, MD 21287, USA

## Abstract

A 17-year-old girl presented with bilious vomiting and abdominal pain to the surgery department. The history was positive for trichotillomania and trichophagia. A CT scan showed a mass in the stomach, which was highly suspicious for a gastric bezoar. Drooping parts of the bezoar caused a duodenal obstruction with secondary acute pancreatitis. The bezoar was removed via a laparoscopically performed gastrotomy.

## 1. Background

This case describes an unusual genesis for acute pancreatitis especially in young women. Rapunzel syndrome is a very rare cause of pancreatitis but should be considered as a differential diagnosis in patients with a significant psychiatric history. This case report presents a novel minimally invasive surgical approach to the treatment of obstructive bezoars causing pancreatitis.

## 2. Case Presentation

A 17-year-old Caucasian female was referred to the surgery department by her primary care physician with a 3-week history of intermittent bilious vomiting and mid-epigastric to right upper quadrant abdominal pain. The pain had started during traveling in her school holidays. In the previous weeks she was admitted to the emergency department for dehydration twice and was released without a definitive diagnosis. On presentation, she had a weight loss of 10 pounds, a decreased appetite, and a reduced urine output. The remainder of the review of systems was negative.

After further extensive inquiry the mother made a side note that the patient had a habit of chewing her hair. Nevertheless she had no signs of alopecia. Furthermore there was a history of swallowing large amounts of gum in her childhood. Otherwise her past medical history was significant for dysmenorrhea which she took oral contraceptives for. The patient was living with her parents and sister and denied any substance abuse. The family history was positive for an unknown clotting disorder, which was noncontributory for the course of this case.

## 3. Investigations

**Figure 1:**
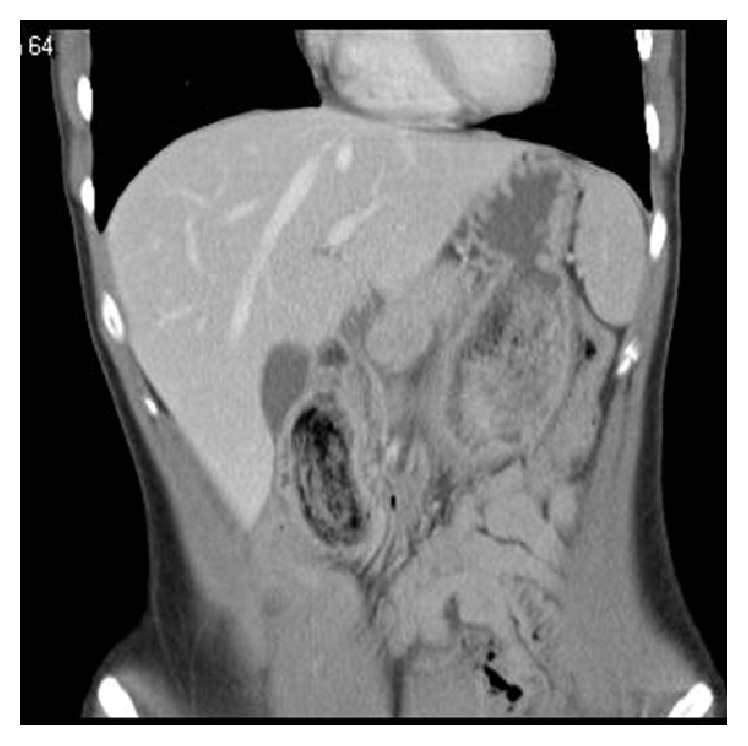
CT image showing bezoar extension from stomach into duodenum.

**Figure 2:**
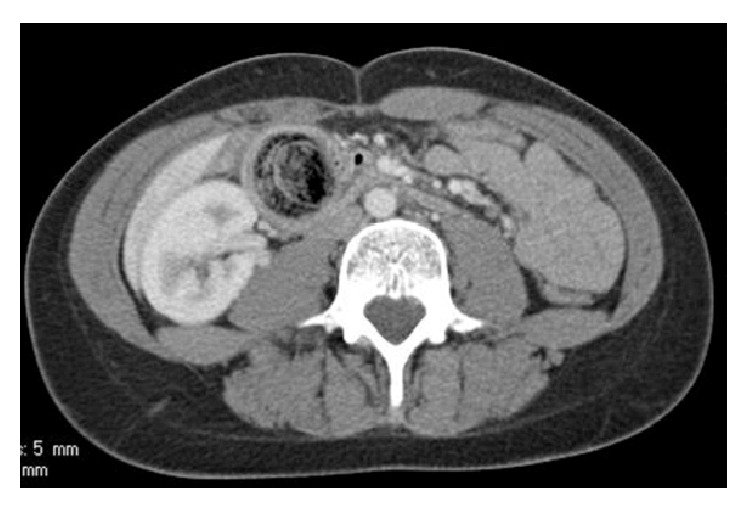
CT image showing bezoar extension from stomach into duodenum.

The patient's lab studies showed elevated markers for pancreatitis. Amylase was 396 and lipase was 639. Her basic metabolic profile was within normal limits. Her complete blood count showed a mild leukocytosis of 11.6. Her Ranson criteria score on admission was 1 (mild pancreatitis). Her radiographic studies were performed including an abdominal X-ray, HIDA scan, and CT scan. The abdominal X-ray merely showed a paucity of gas. The CT scan of the abdomen however revealed a mass, which was considered as a possible trichobezoar in the stomach and proximal duodenum. This bezoar could strongly be related to the history of trichophagia and would be a cause of duodenal obstruction with secondary pancreatitis, which was detectable on CT imaging (Figures 1 and 2).

## 4. Treatment

**Figure 3:**
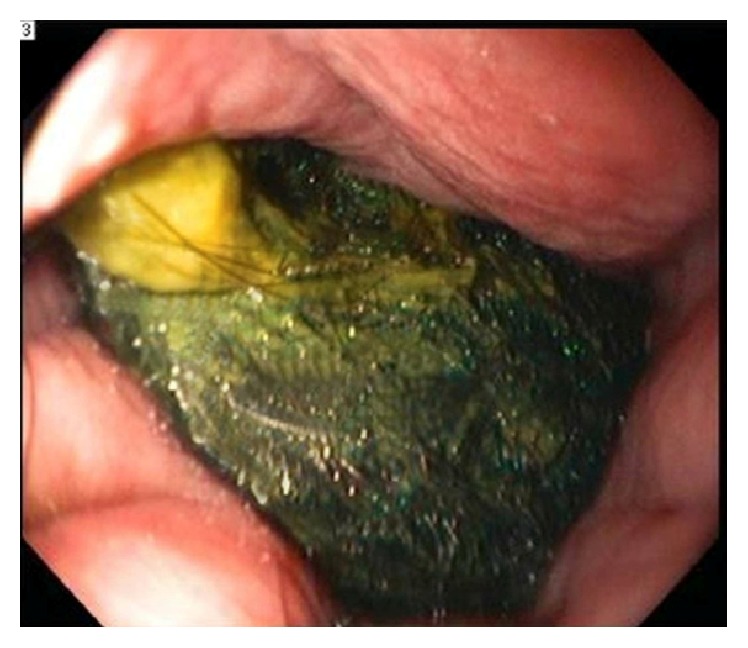
Endoscopic image of bezoar in stomach causing complete occlusion of duodenum.

At first the patient was treated conservatively. She was placed on IV fluids and received NG tube, antiemetics, and analgesia. Then the gastroenterologist attempted to do an EGD to evaluate the mass. The EGD revealed a distal esophagitis, gastritis, and trichobezoars occupying and obstructing the lumen of the stomach. The duodenum could not be intubated due to the mass (Figure 3); therefore the gastroenterologist was unable to evacuate the trichobezoars endoscopically.

**Figure 4:**
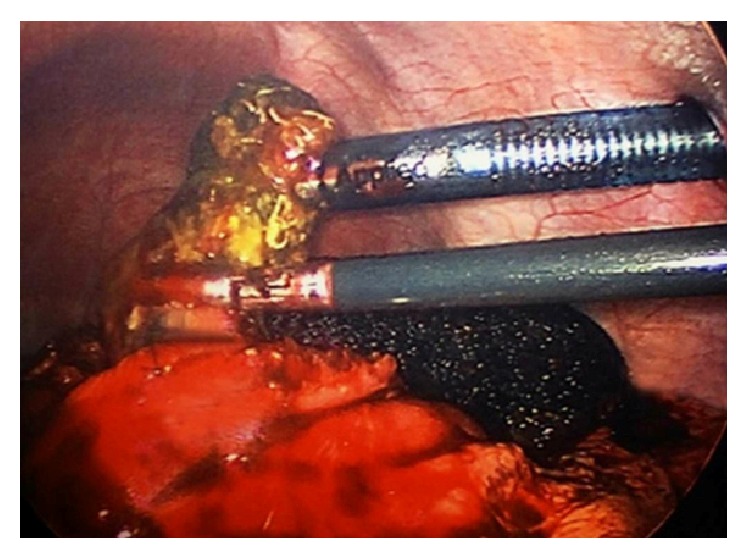
Gastrotomy incision.

Surgery was consulted for operative intervention. The decision to undergo a laparoscopic gastrotomy with exploration and removal of the bezoar in her stomach and duodenum was made. The procedure was performed under general anesthesia without any complications. Four ports were used. After identifying the stomach and feeling a bulge in the distal part of it, an incision for the gastrotomy was made with scissors and electrocautery (Figure 4).

**Figure 5:**
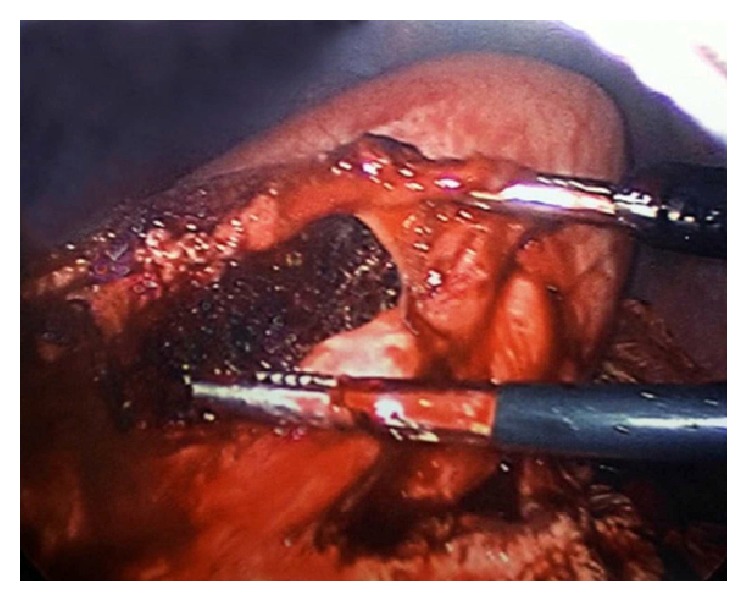
Piecemeal extraction of the bezoars from gastrotomy incision.

A large bezoar was found in one part of the stomach, attached to the pylorus, and another part was extending into the duodenum. After detaching the bezoar from the stomach mould, it was divided into 3 pieces in order to extract it from the port sites (Figure 5). These parts were removed with an Endocatch*™* through the umbilical incision. The gastrotomy site was closed with Endostitches*™* vicryl sutures. An endoleak test was performed with air insufflations via an intraoperative EGD. The total operation time was 2.5 hours.

The patient tolerated the procedure well without any complications. Prior to discharge the patient's pain was adequately controlled with p.o. narcotic pain medication. The follow-up radiographic studies showed no evidence of leakage or signs of obstruction or free air. She was discharged with a stool regimen and instructions for a follow-up as an outpatient.

## 5. Outcome and Follow-Up

One month after surgery the patient was gaining weight and had no nausea or vomiting or any abdominal symptoms. Her wounds had also completely healed up. Furthermore we planned an outpatient follow-up and psychotherapy for prevention of recurrences, which present in 20% of the cases [1].

## 6. Discussion

“Rapunzel syndrome” is a unique manifestation of an advanced gastric trichobezoar, which was first described in literature by Vaughan et al. in 1968 and has three common features [2, 3]:

- A trichobezoar in the stomach.
- A long “tail” of hair strands that extends through the pylorus into the small intestine and sometimes even into the large intestine.
- Gastrointestinal symptoms.

The complications associated are bleeding, perforations with peritonitis, ulcers, enteropathy with protein loss, appendicitis, and, in severe cases like ours, pancreatitis [4–7].

At this point of time approximately 30 cases of Rapunzel syndrome have been published worldwide [8–10]. The first publication of a bezoar was in 1779 by Baudamant, who made the discovery during an autopsy [11]. Schönborn reported the first surgical intervention in 1883 [12]. Similar to our case, trichobezoars are most often being observed in young female patients with long hair, in many cases associated with the psychiatric disorders trichotillomania and trichophagia [13, 14]. As per ICD-10 (F63.3) trichotillomania is classified as an impulse control disorder, characterized by repetitive, compulsive urge to pull out one's hair leading to noticeable hair loss [15]. However many of these patients ingest not only their own hair, but also the hair of other humans and animals, rugs, and synthetic or natural fibers [16, 17].

It is assumed that the slippery nature of hair and its entrapment within the gastric folds could be the reason for cross-linkage and the formation of bezoars [18]. The symptoms develop slowly and may go unnoticed, what explains the extraordinary size of the bezoars, which can almost form a corrosion cast of the stomach at the time of the diagnosis. Patients initially present with unspecific abdominal pain (37%) and weight loss and anorexia (38%). Later during the course the chief complaints are nausea/postprandial vomiting (33%) and intermittent abdominal cramps [19–21]. The physical exam often shows a palpable mobile epigastric tumor (70%). The additional finding of diffuse alopecia and changes in the scalp skin, eyelids, or eyebrows manifests the diagnosis [22, 23].

The objective of the treatment is the mechanical removal of the trichobezoar and the prevention of recurrences with psychotherapy [1, 24, 25]. The extraction of the mass can be done either endoscopically or surgically with laparotomy or laparoscopy [1]. In a retrospective review of 7 cases of trichobezoar of which 5 were diagnosed with Rapunzel syndrome, all patients required an exploratory laparotomy for definitive treatment [26]. Our approach however was a laparoscopic gastrotomy with exploration and removal of the foreign body. A laparoscopic removal of a gastrointestinal bezoar was first performed by Nirasawa in 1998, but this was not a Rapunzel syndrome by definition [24].

The treatment of Rapunzel syndrome is still the subject of an ongoing debate, since a gold standard does not exist. A retrospective review of laparoscopic versus open bezoar removal by Yau et al. shows that laparoscopic removal has a lower rate of complications. Of note, these were not Rapunzel syndromes. In our literature review we only found 3 other reports of successful laparoscopic management of Rapunzel syndrome [27–29]. Dorn et al. describe a technique that involves an intragastric port, which is used to remove the bezoar in a piecemeal technique to minimize spillage. However it was associated with prolonged operative time (6 hours). Our approach is close to Kohler's technique with gastrotomy and an Endocatch-piecemeal removal of the bezoar. Although it is associated with greater spillage potential, our patient did not have any infection-related complications postoperatively. Furthermore the operative time was significantly shorter.

Reasons for a laparoscopic approach are most notably attributable to the epidemiology of this condition. Since the common presentation of trichobezoars is in young females, attention should inter alia be paid to the aesthetic result, which speaks in favour of a laparoscopy (less scarring, easier postoperative management, and shorter convalescence time) [30]. Furthermore it should be considered that a laparotomy scar could cause an additional psychological burden to patients with a psychiatric history. That is why in our opinion the laparoscopic management of Rapunzel syndrome is safe and effective and should be preferred to an open approach in young patients with bezoars.

## Additional Points

The learning points are as follows:

- Rapunzel syndrome is a rare condition but should be included in differential diagnosis in young patients with pancreatitis.
- Laparoscopic management of a gastric bezoar-induced duodenum obstruction with secondary pancreatitis is safe and effective.
- A detailed anamnesis in the assessment is significant and often leads to the diagnosis.
